# Treatment of Loose Cartilage in the Knee Joint

**Published:** 1902-04-19

**Authors:** 


### TREATMENT OF LOOSE CARTILAGE IN THE KNEE JOINT.

Although in many cases of derangement of the internal structures of the knee-joint, the function of the joint may be restored by replacement of the cartilages by manipulation, followed by rest; this is a result which cannot always be counted on; and even when an apparent cure has been effected relapses are in many cases apt to occur from such small causes that it becomes necessary to seek relief from operative treatment. In recording the details of a long series of such cases Mayo Robson[1] points out that the attempts which are sometimes made in the course of operations to fix the torn and displaced cartilage in position are not necessary, it being quite sufficient to cut off the loose portion. By so doing the operation is much simplified, and the chances of infection of the joint are greatly diminished. The operation, he says, is one of the simplest. An oblique incision is made from the inner border of the lower end of the patella downwards and backwards, down to and through the synovial membrane, so as to open the joint freely without dividing either the ligamentum patellæ or the internal lateral ligament of the knee. If the external ligament be the one which is displaced, then the incision is to be made on the outer side of the joint. On retracting the edges of the incision and flexing the knee the displacement at once becomes manifest. The loose portion of cartilage is seized and snipped off by means of scissors, and then the synovial membrane, the capsule of the knee, and the skin are separately sutured. It is both unnecessary and inadvisable to insert the finger into the joint or to syringe the articular cavity. Needless to say, the strictest asepsis must be secured. For several years Mayo Robson has been accustomed to cut away the fractured displaced cartilage, which is frequently so distorted as to no longer represent the semilunar pad that once served a useful purpose, and a sufficient time has now elapsed, he says, to enable him to speak with confidence as to the results; for in no case has any inconvenience been experienced by the loss of the cartilage, and the functions of the knee-joint have on recovery been as perfect as before the accident by which the disability had been originally caused.

[1] Brit. Med. Journ., April 12.

